# “Moments of Clarity”: A Qualitative Study to Understand Factors Protecting Patients in Active Drug Rehab from Deaths of Despair

**DOI:** 10.1177/23743735261422278

**Published:** 2026-02-25

**Authors:** Kaléi H. Crimi, Kristin Cunningham, Lisa Albert, Pauline Hwang, Michael Evans, Heather Stuckey, Daniel R. George, Emily Brignone, Judith Hupcey, Jennifer Kraschnewski

**Affiliations:** 1 8082 The Wright Center for Community Health, Administration, Scranton, PA, USA; 2 Ross and Carol Nese College of Nursing, 8082 Penn State University, University Park, PA, USA; 3 12310 Penn State University College of Medicine, Hershey, PA, USA; 4 575429 Highmark Health, Pittsburgh, PA, USA

**Keywords:** mental health, social determinants of health, rural health, addiction, qualitative methods, disease of despair, drug recovery

## Abstract

Over the past three decades, US mortality rates resulting from suicide, drug overdose, and alcohol-related liver disease—collectively referred to as Deaths of Despair (DoD)—have risen sharply. While epidemiologic patterns are well documented, less is known about the lived experiences of those vulnerable to DoD and the factors that support recovery. This study partnered with an addiction recovery center in a central Pennsylvania community disproportionately affected by despair-related conditions. Seventeen individuals in active recovery participated in in-depth interviews to explore pathways leading to despair and addiction, and sources of resilience. Thematic analysis using MAXQDA software identified three primary themes with subthemes: barriers to recovery (grief from personal loss, isolation, and ineffective coping with pain), shifts from despair (ie, “moments of clarity”) (change in motivation, remorse, and embarrassment), and recovery support (higher power and family, community and healthcare support). These findings provide qualitative insight into the biopsychosocial dynamics of despair and recovery and highlight the importance of grief support, coordinated pain management, emotional reflection, and social and spiritual resources. Such insights may inform clinical practice, community programs, and policy interventions aimed at reducing DoD and supporting long-term recovery.

## Introduction

Deaths of Despair (DoD), defined as mortality from suicide, drug overdose, and alcohol-related liver disease, have risen steadily in the US over the last three decades affecting a diverse range of populations.^1,2^ In 2021, 209 225 annual despair-related deaths were documented, with the sharpest increases in the South, West, and rural regions of the US.^3^ Adults aged 45-64 have experienced the highest DoD rates, particularly for drug and alcohol-related deaths, with a higher incidence among those facing chronic health conditions, disability, job loss, or social isolation.^3,4^ The COVID-19 pandemic further accelerated DoD trends, particularly overdose and alcohol-related mortality.^5^

Although initially identified among rural, working-class, white men and women with low educational attainment,^1^ DoD has recently increased for indigenous persons and Black Americans.^6,7^ In part due to rising DoD, US life expectancy is at its lowest level since 1996^4^ and declining relative to other high-income countries,^8^ with mortality differentials especially pronounced for lower income groups.^9^ Indeed, the US premature death rate is nearly double that of comparable nations, and DoD—along with chronic disease and the lingering effects of the COVID-19 pandemic—accounts for 12% of the premature death rate.^10^

DoD have been most prevalent in regions marked by long-term labor market decline and among working-class cohorts.^11^ It is theorized that changing macroeconomic conditions have weakened family structures and reduced access to high-quality health care, causing social fragmentation, loneliness, and loss of future-oriented hope.^12–15^ At the individual level, these causal processes are believed to elevate the risk for biopsychosocial changes (eg, chronic pain, anxiety, depression) that heighten risk for self-harm and substance use illnesses.^10^ Rising access to opioids, cheap alcohol, and firearms within an environment of rising despair has created dangerous and potentially lethal conditions for those at risk. Cross-national comparisons suggest that the US's limited social safety nets also play a causal role in despair-related mortality—ie, other wealthy nations exposed to similar macroeconomic conditions have avoided comparable rises in DoD, likely due to stronger social protections that buffer individuals from despair and support human flourishing across the life course (eg, prenatal and maternal care, preschool, low-tuition higher ed, mandatory vacation time, etc).^12^

While much research has examined the epidemiology and theoretical causes of DoD, less attention has been paid to the lived experience of despair, particularly in individuals with a history of substance abuse, and ways it might be mitigated or overcome.^14–17^ Recovery from substance use disorder is increasingly understood as a long-term, non-linear, evolving process, often requiring multiple attempts before sustained stability is achieved.^18^ While approximately 75% of individuals who experience addiction ultimately recover over time, relapse rates remain high (40%-60%),^19^ with premature treatment dropout further compounding risk.^20^ Negative emotions—including despair, guilt, shame, and anxiety—as well as exposure to stress and environmental cues are central risk factors for relapse,^21^ underscoring the importance of understanding what motivates and sustains recovery.

The purpose of this study was to build on prior research^22–24^ to explore the lived experiences of individuals in economically and socially vulnerable communities who are actively engaged in recovery and at high risk for DoD. Specifically, we sought to identify the personal, interpersonal, and structural factors that participants perceived as critical for finding clarity, sustaining motivation, and maintaining abstinence. We conceptualized recovery as a dynamic process through which individuals improve their health and wellness, pursue self-directed lives, and strive toward their full potential.^25,26^

## Methods

### Study Population and Recruitment for Interviews

We previously partnered with Highmark Inc., a large U.S.-based health insurance company, to identify DoD hotspots in Pennsylvania,^24^ which allowed for deeper insight into the social context of despair-related illness. This data included de-identified diagnoses and addresses for members of employer-sponsored Affordable Care Act and Medicare insurance plans during 2018. A residential addiction treatment center was identified within one of the hotspot zones, and we engaged the organization's leadership as a collaborator. The Pennsylvania State University's Institutional Review Board approved all procedures.

Staff at the residential addiction treatment center helped identify potential participants from their residential community. The purposive sample was 1) > 45 years of age (due to this cohort being identified as especially high risk in prior studies), 2) English-speaking, and 3) in active addiction recovery. Demographic information was documented and collected and verbal consent for participation was obtained before the initial interview. Upon completion of the interview, participants received a $50 gift card.

### Qualitative Data Collection

**Figure 1.**
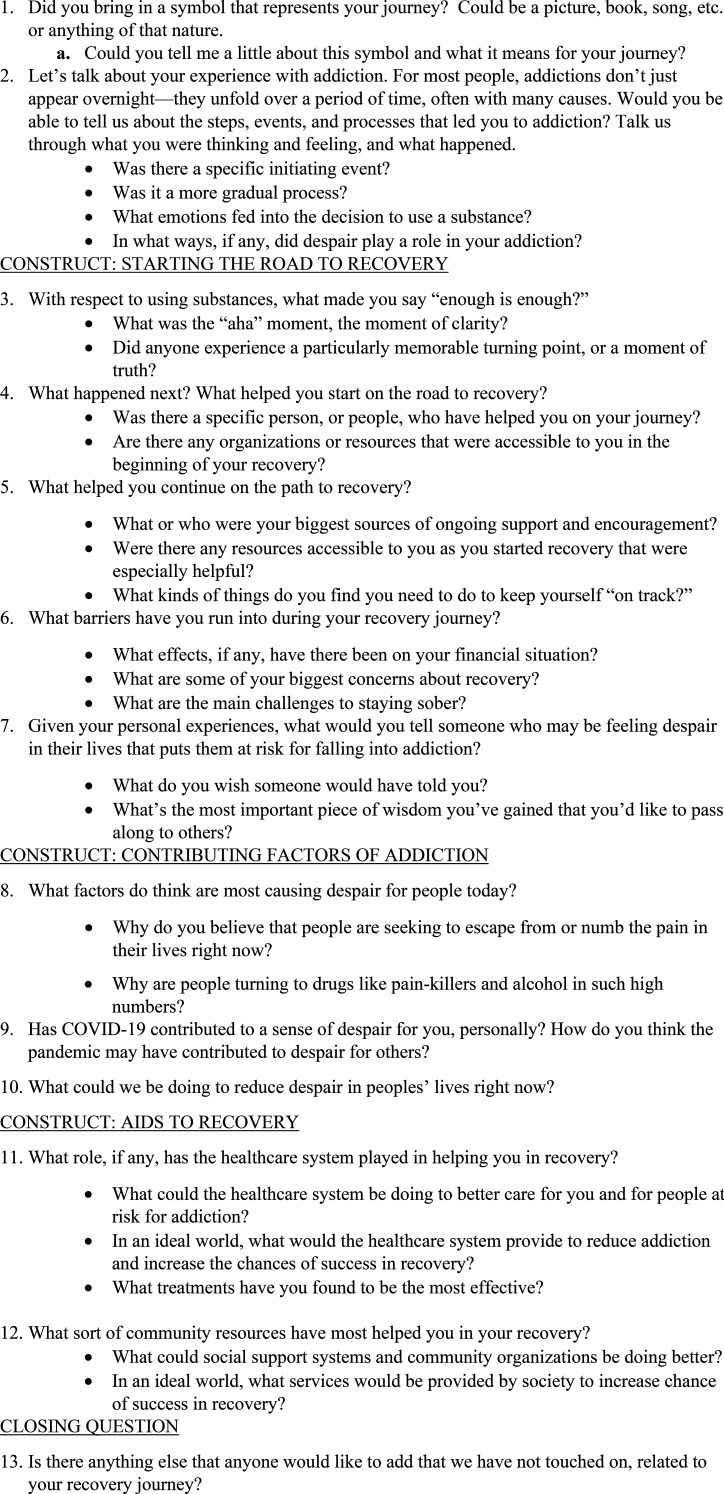
Semi-structured interview guide.

Semi-structured interviews were held from 12/2022 to 03/2023 using an interview guide (see Figure 1) developed following a review of the literature. The guide explored three concepts: 1) general awareness and beliefs about the recovery journey, 2) contributing factors of addiction, and 3) aids to recovery. The interview guide was pilot tested with four research team members (LA, PH, KC, ME), and the interviewers completed two recorded practice interviews to ensure consistency in the interview process.

### Thematic Analysis (Using in Vivo Coding)

Audio recordings of the interviews were transcribed verbatim and de-identified. The analytic team was comprised of three nursing faculty (KC, PH, LA) and a qualitative methodologist (HS). An In Vivo approach to coding allowed researchers to capture short phrases from the actual language in the qualitative data so that terms used by the participants could be described.^27^ After each sentence in the first round of coding, an In Vivo code was placed in quotation marks, and each sentence of data received its code, using the participant's words. A qualitative software program designed to manage codes and data, MAXQDA, was used to assist in In Vivo coding by permitting the researchers to select a word or small phrase from the data and assigning the selected text as an In Vivo code. In the second round, researchers condensed the number of In Vivo codes to group similar codes. Those codes that were participants’ words were kept in quotation marks, and those that were condensed by the researcher were not. Memos were also used when condensing the codes to trace and reflect on the coding process. This preliminary codebook was created by two coders (KC and PH) and the qualitative methodologist (HS).

As coding occurred, these individuals also checked for saturation, which was achieved between transcripts 9 and 10. Data collection continued until coding saturation was reached, indicated by redundancy in responses and no emergence of new codes across subsequent interviews occurred. The researchers then independently re-coded the first two transcripts to determine whether the participant data could be appropriately assigned to the codes. Cohen's Kappa was used to assess inter-rater reliability between the two coders, and a score of 0.77 (moderate) was achieved. The remaining data transcripts were divided and coded by one analyst (KC). Throughout the process, coding meetings were held to discuss discrepancies iteratively; all codes were adjudicated by a third researcher (HS). All investigators (authors of this paper) reviewed the coding patterns/frequencies that informed the grouping of codes and contributed to the development of broader themes, finding patterns that connected related concepts.

## Results

### Participant Characteristics

Seventeen interviews were conducted, averaging 50 min. Of the 17 participants, the majority were White (52%), Male (82%), Divorced (59%), with a mean age of 59.5 years. Most (83%) had completed high school with some college or technical training. Although 65% were currently unemployed, 59% reported having health insurance, and all had been in active recovery for less than a year (see Table 1).

**Table 1.** Demographic Characteristics (n = 17).

Characteristic	Category	n (%)
Age	Mean (SD)	59.5 (±3.4)
	Range	55–67
Gender	Male	14 (82%)
	Female	3 (18%)
Race/Ethnicity	White/Caucasian	9 (53%)
	Black/African American	5 (29%)
	Biracial (Black-Hispanic)	1 (6%)
	Hispanic/Latino	1 (6%)
	Native American	1 (6%)
Highest education level*	Middle school	2 (12%)
	Some high school	1 (6%)
	High school graduate	5 (29%)
	Some college	4 (24%)
	Technical/professional school	2 (12%)
	College degree	3 (18%)
Marital status*	Never married/single	4 (24%)
	Married	2 (12%)
	Divorced/separated	10 (59%)
	Widowed	1 (6%)
Children	Mean (SD)	1.5 (±1.6)
	Range	0–6
Health insurance status	Insured	10 (59%)
	Uninsured	6 (35%)
	Unsure	1 (6%)
Employment status	Employed	5 (29%)
	Unemployed	11 (65%)
	Long-term disability	1 (6%)
Years in recovery	<1 year	17 (100%)
	10–15 years	0 (0%)

* The total category percent may not add up to exactly 100% due to rounding to the nearest whole number.

### Themes

Analysis identified three main themes—barriers to recovery, shift from despair, and recovery support, each with associated subthemes. Themes and subthemes are illustrated in Table 2.

**Table 2.** Thematic Table.

Themes	Subthemes
Barriers to recovery	Grief from personal loss
	Isolation
	Ineffective coping with pain
Shift from despair (ie, “moments of clarity”)	Change in motivation
	Regret and embarrassment
Recovery support	Higher power support
	Family support
	Community support

#### Theme 1: Barriers to Recovery

Nearly all participants traced their escalation from social substance use to addiction, often linking relapse to grief, isolation, or ineffective coping with pain.

##### Grief from Personal Loss

Losses such as the death of a parent, sibling, or child, as well as divorce, were common catalysts for substance use and addiction. Participants described these events as destabilizing inflection points for despair that created coping challenges and drove them toward substances that provided numbing or escape.

Me and my wife separated, and I started drinking pretty heavy. And I've been drinking pretty much ever since. [P 11]

The death of a father was a recurring theme, and was frequently linked to relapse:

… if he (father) would’ve been alive, none of this (addiction) would’ve happened. [P 7]

##### Isolation

Isolation and resulting loneliness were cited as recurring drivers of despair and relapse, with participants describing a tendency toward withdrawal:

[After leaving the recovery house too soon], I had a lot of downtime, I started smoking weed again. [P3]

The COVID-19 pandemic also compounded isolation. As one participant explained:

[I] didn’t want the germs … didn’t want to get sick by being out in public. [P 8]

##### Ineffective Coping with Pain

Physical changes from illness or injury, especially those causing biopsychosocial pain, or perceived pain in a state of despair, placed individuals in states of distress and often led to an escalation of substance use or relapse. A veteran, injured during service, spoke of losing a job and its related anchoring in a sense of purpose, as well as chronic pain from the injury:

Once I couldn't get no more of the pharmaceutical medications that I preferred, I went back to, with the resentment that I got hurt over the service, that it shouldn't have happened to me. And then it really, really took off. [P 9]

Similarly, a factory worker who had injured his hand, resulting in a permanent deformity, loss of use, and chronic pain, told a similar story of despair and escalation of substance use:

During treatment they're giving me oxycodone for pain. I'm getting five, six different drugs. I'm there 32 days. [I refused therapy] because now I'm hooked on these things … I'm buying pills illegally because I still want them. I'm just laying in that bed all day, woe is me. [P 17]

Pain from mental health conditions presented challenges in coping, and failure to successfully manage these issues was a recurring cause of relapse. Gaps in the healthcare system also played a role for some patients. For example, after a release from prison, a participant went through health insurance coverage gap issues, leaving him without therapy and medication to assist in keeping despair at bay:

And I started getting depressed. The anxiety started getting bad, and yeah, I ignored that. And it was a major factor in why I started using again. [P 5]

#### Theme 2: Shift from Despair (ie, “Moments of Clarity”)

Despite barriers, participants consistently described decisive mindset shifts that reoriented them toward recovery—revelatory “moments of clarity” that changed their relationship to despair and transformed the course of their lives.

##### Change in Motivation

A key component of this shift involved participants finding renewed motivation that ushered in a sober way of thinking. Participants described it in terms of stepping out of feelings of despair into a new willful commitment to recovery:

So, what made me change my mind and come back here today is I decided I want to live. No matter what now, I want to live. [P 1]

Participants also found purpose in helping others, reframing recovery as an opportunity for contribution. Changing this internal disposition established a healthier orientation to the future and anchored recovery in personal commitments and accountability:

I have a lot to offer and I think, I know, that helping other people will help me stay clean and sober and that's what I want to do. [P 6]

Sensing a pathway out of despair also provided a sustaining source of strength that participants found emboldening:

I’m ready to take on the new challenge of a new day. Every day. What's different this time … is that for the first time in my life, I’m not terrified by the thought of never being able to get high again, get drunk or high again … today, it's knowing it's something better to live for. When you taste recovery, even though I only tasted for two years, that's a good taste. [P 1]

##### Remorse and Embarrassment

Along with an emergent vision of a clean, purposeful, and future-oriented life, participants achieved clarity by accepting that their previous choices had harmed relationships with loved ones. Confronting remorse and coming to terms with the embarrassment imposed on their families was sufficient motivation to take steps away from living in addiction:

The pain that I caused myself and the pain I seen in my wife face and my children's faces. I got tired of saying, ‘I'm sorry’. [P 7]

By bottoming out and confronting their worst internal aspects, participants found internal motivation to no longer be defined by the despair and emptiness underlying their substance use:

Living the life of an addict … it was terrible. You don't care. You don't care for yourself. You don't– you don't love yourself. And anybody out there, you really don't– you don't care. You just don't– you just– your whole morals and values, and everything is gone. [P 6]

#### Theme 3: Recovery Support

Sustained recovery was linked to support systems, which participants described across three domains: a higher power, family, and community/healthcare resources.

##### Higher Support

For some, faith and spirituality, ie, a belief in a higher power, provided a practical and conceptual support system. Feeling a connection with God via spiritual traditions provided safety and comfort during times of despair and addiction, offered a source of hope for a better future, and made accessible a continuous and reliable support:

[God] was always there … the extra level of support*.* [P 13].

I realize it's something that I most definitely can't do by myself … and thanks to my God, Jehovah, that he helped me endure. He kept me—he protected me while I was out here in this addiction. And I still was praying to him to get out of it. [P 1]

##### Community and Healthcare Support

Participants also identified community- and healthcare-based resources that supported their recovery journey, in addition to their current rehabilitation program. Alcoholics Anonymous (AA) and Narcotics Anonymous (NA) were both viewed as vital:

The 12-step fellowship gives me a gauntlet of experience. That's why I go. It's important. Plus, it worked. It worked for me. It makes me a better person. [P 12]

##### Family

Family relationships, where intact, were also important sources of motivation and accountability in maintaining sobriety:

And I got my mom and my brother. They're in Southern California, but they're on my side at least in terms of being moral support. [P 8]

As with a higher power, supportive organizations, and family connection required participants to embrace the humility of being unable to tackle recovery fully on their own while seeking community and fellowship in others.

## Discussion

This study deepens prior qualitative work on DoD by illuminating how the structural and emotional factors driving the crisis are experienced at the individual level. Our findings provide insights into the lived experience of despair and highlight the biopsychosocial dynamics that contribute to addiction and support recovery. Unhealthy grieving emerged as a common triggering event for addiction and relapse, underscoring the need for policies and programs that support individuals navigating loss, especially in rural or underserved areas.^20^ Physical injuries and chronic pain were also key catalysts, pointing to the importance of coordinated care for individuals prescribed opioids—care that should include deprescribing plans, risk screening for dependency risk factors, and access to alternative pain management resources, especially in settings with high rates of work-related injuries.^25^ These individual experiences echo broader macroeconomic and healthcare gaps described in the DoD literature.

A second major finding was the “moment of clarity” participants described as a turning point in recovery. These internal wake-up calls profoundly shifted individuals away from despair and toward sobriety. Consistent with prior research, intrinsic motivation was central to developing long-term coping skills and achieving abstinence and maintaining long-term goals, such as active recovery,^19,23–26,28,29^ but our study adds nuance by showing how helping others, reflecting on past harm, and reclaiming a sense of social usefulness can provide durable sources of resilience. Such shifts may interrupt pathways toward despair-related mortality, particularly when reinforced by supportive infrastructure.^30,31^

Remorse and embarrassment also emerged as important emotional inflection points. Confronting the pain caused to loved ones marked a turning point, shifting participants from a state of emptiness and despair toward a renewed sense of purpose and the beginning of their recovery journey. Such experiences of remorse not only heightened self-awareness but also fostered an internal desire to change, aligning closely with the intrinsic motivation that supports sustained recovery. This aligns with prior research showing that emotions like remorse, guilt, and shame can prompt moral reflection and motivate reparative behavior when individuals perceive a viable path to redemption.^31^ Access to mental health professionals and recovery coaches who can support this emotional work may help individuals process these emotions and strengthen recovery trajectories.

Finally, participants emphasized the role of recovery supports—spiritual belief, family, and 12-step programs such as AA/NA—in maintaining sobriety and rebuilding identity. These supports buffered against isolation and hopelessness, consistent with prior findings.^19,23,29–31^ Investment in social and spiritual infrastructure may be especially important in regions disproportionately affected by DoD.

### Limitations

This study has several key limitations. The research was conducted at a single residential addiction treatment center in a rural community setting. Thus, findings may not reflect experiences in urban areas or in populations without comparable social, institutional, and other supports. The sample was relatively small, largely White, English-speaking, and over age 45, restricting diversity of perspectives. Participants were also in active recovery; as such, their insights may not capture the experiences of those without access to treatment or those at earlier stages of addiction. These factors limit transferability but provide context for understanding the study's scope.

### Strengths

Despite these limitations, the study has several strengths. All participants were from a region (Appalachia) and age group disproportionately affected by DoD and premature mortality. The focus on individuals in active recovery at high risk of DoD offers valuable insight into resilience and transformation. Methodological rigor in interviews and analysis adds qualitative richness to our understanding of despair and recovery.

## Conclusion

This study highlights the lived experiences of despair and recovery, including the roles of grief, pain, emotional reflection, moments of clarity, and recovery supports. Future research should explore these dynamics across more diverse populations and settings, including Indigenous and Black communities who are increasingly affected by DoD. Expanding this work will help identify and evaluate effective interventions—clinical, social, and policy-based—that can strengthen recovery infrastructure and mitigate despair. Addressing the DoD crisis requires investment at local, state, and national levels to reduce mortality and foster human flourishing.
